# Society Meetings

**Published:** 1895-05

**Authors:** 


### Society Meetings.

The Third International Congress of Dermatology will be held in London, August 4 to 8 inclusive, 1896, under the presidency of Mr. Jonathan Hutchinson. The vice-presidents for the United States are: Dr. Duhring, of Philadelphia; Dr. White, of Boston; Dr. Hyde, of Chicago; Dr. Bulkley, Dr. Keyes and Dr. Fox, of New York. The treasurer is Mr. Malcom Morris. Dr. J. J. Pringle, 23 Lower Seymour street, London, W., is the secretary-general and Dr. George Thomas Jackson, of 14 East Thirty-first street, New York, is secretary for the United States.

The following are the regulations of the congress:

1. All duly qualified medical men, British or foreign, or others interested in science invited by the council, who shall have paid the fee of £1[1] sterling, and who shall have enrolled themselves shall be members of the congress and entitled to the volume of transactions.

2. The official languages of the congress shall be English, French and German, but with the permission of the president, members may express themselves in the language in which they are most familiar.

3. The proceedings of the congress shall be embodied in a volume of transactions, edited by the executive council.

4. Communications relative to membership, papers or other matters connected with the congress, should be addressed to the secretary-general, Dr. J. J. Pringle, 23 Lower Seymour street, London, W., or to one of the foreign secretaries.

5. The fee for membership shall be payable in London at or before the opening of the congress.

It will greatly facilitate the work of the executive if the fee is forwarded as soon as possible after the 1st of May, 1896.

6. Members who are unable to attend the congress shall receive the volume of transactions.

7. The subjects treated of shall be of two orders :

(1) Those selected beforehand by the executive council and introduced by gentlemen chosen for that purpose by the council.

(2) Those selected by individual members themselves.

8. Subjects selected for debate by the council shall take precedence over those selected by the members.

9. The sittings of the congress shall take place from 11 to 1 in the forenoon and from 3 to 5 in the afternoon of each day.

10. There shall be clinical demonstrations of patients every morning from 9 to 10.30 and every afternoon from 2 to 3.

11. Members contributing papers must submit an abstract of them to the secretary-general on or before the 1st of May, 1896, which will be printed either in full or in part and embodied in the general program of the congress which will be distributed at its opening.

12. At every debate precedence will be given to gentlemen who have communicated beforehand their intention to take part in it.

13. No papers lasting more than twenty minutes will be permitted. Speeches will be strictly limited to ten minutes each.

1. The equivalent of £1 sterling is : French, 25 francs ; German, 20 marks : Italian, 25 lire ; American, $5.

Manuscript of the papers read must be left with the secretary-general before the end of the sitting. The executive council shall decide as to the entire or partial publication of such papers in the transactions of the congress.

---

The Ohio State Medical Society will hold its next annual meeting at Columbus, May 15, 16 and 17, 1895. This will also be its semi-centennial anniversary, which the society proposes to celebrate in a befitting manner. An interesting program is preparing, and members of the profession from other states are cordially invited to attend. The president of the society is Dr. N. Kinsman, of Columbus, and the secretary is Dr. Thomas Hubbard, of Toledo.

---

The American Medical Publishers' Association will hold its second annual meeting at the Eutau House, Baltimore, May 6, 1895. Dr. Landon B. Edwards, of Richmond, is president and Mr. Charles Wood Fassett, of St. Joseph, is secretary. Medical editors, publishers and business managers are cordially invited to attend the meeting.

---

The American Medical Association will hold its forty-sixth annual meeting at Baltimore, May 7th, 8th, 9th and 10th, under the presidency of Dr. Donald Maclean, of Detroit. Dr. George H. Rohé, of Baltimore, is assistant secretary and chairman of the committee on exhibits.

---

The Association of Military Surgeons of the United States, as previously announced in the Journal, will hold its next annual meeting at Buffalo, Tuesday, Wednesday and Thursday, May 21, 22 and 23, 1895, under the presidency of Brigadier-General George M. Sternberg, surgeon-general of the U. S. Army. The object of the association is to promote and develop military medicine, surgery and hygiene in every possible way. Membership is open to all commissioned medical officers in the service of the United States or of the several states and territories upon the payment of $5, annual dues.

The association, it will be observed, is not hampered by the code of ethics, but welcomes scientific men who are in the public services. It has now 250 members residing in thirty-five states. Applications for membership should be made to the secretary, Lieutenant-Colonel Chancellor, M. D., 515 Olive street, St. Louis,

Mo., giving full name, rank and address and enclosing fee of $5.00.

Major A. H. Briggs, M. D., surgeon of the 65th Regiment, N. G., S. N. Y., 267 Hudson street, Buffalo, is chairman of the committee of arrangements. Dr. Briggs is an executive officer of skill and capacity and under his guidance elaborate preparations are making for the entertainment of the visitors. The headquarters of the committee have been established at the Hotel Iroquois and a number of sub-committees have been appointed.

Subscriptions for the entertainment of the guests are solicited and these should be addressed to Dr. A. A. Hubbell, chairman of the finance committee. Public-spirited citizens should not be slow in sending in their contributions and thus indicate their pride in Buffalo and a desire for its prosperity.

The scientific program was published in the April number of the JOURNAL. The sessions will be held at the Star Theatre.

---

THE Medico-Legal Society announces that it will hold a medico-legal congress at or near the City of New York during the last week of August or first week of September, 1895, (time and place to be hereafter announced,) open to all students of medical jurisprudence, under the charge of a committee of arrangements, composed as follows: Ex-Surrogate Rastus S. Ransom, chairman; Clark Bell, Esq., secretary; George Chaffee, M. D., treasurer; Moritz Ellinger, Esq., H. W. Mitchell, M. D., Constantine J. MacGuire, M. D., Prof. A. M. Phelps, M. D.

An enrolling fee of $3 will be required of each member to aid in the expenses and the publication of the bulletin of the congress, to be sent each member free.

1. The department of medico-legal surgery will be under the charge of the officers of the section on medico-legal surgery. Granville P. Conn, chairman, of Concord, N. H.

2. The department of psychology and psychological medicine will be under the charge of the officers of the psychological section. Prof. Elliott Coues, chairman, Washington, D. C., and sub-committees.

(1) Sociology and criminology will be in charge of a sub-committee, of which Hon. Moritz Ellinger will act as chairman.

(2) Legal responsibility of the inebriate will be in charge of a sub-committee, of which T. D. Crothers, of Hartford, Conn., wil- act as chairman.

(3) The chairmen of sub-committees of mental medicine and other psychological and medico-legal branches and departments will be hereafter announced.

3. Microscopy in forensic medicine. Prof. M. C. White, of New Haven, will act as chairman of sub-committee.

4. Chemistry will be in charge of a sub-committee of which Prof. H. A. Mott, LL. D., will act as chairman.

5. Dr. Paul Gibier will act as chairman of the department of bacteriology in medical jurisprudence. The roll of members, the official program, the title of papers and the various officers, committees and sub-committees will be forwarded to all members by circular and announced in the *Medico-Legal Journal* and the lay and medical press.

A general invitation to all persons interested in the science of medical jurisprudence is extended and the members of the society, active, corresponding or honorary, who wish to enroll will forward their names and the enrolling fee to the secretary or treasurer. A considerable number of papers have already been secured and the interest already taken in the movement warrants the expectation of a large and influential meeting of the friends and students of forensic medicine on the occasion.

A formal and official circular will be issued later and all persons contributing papers will please forward their titles to the secretary to enable proper classification to be made for the formal circular.

H. W. Mitchell, M. D., *President.*
Clark Bell, Esq., *Secretary.*

